# Seedling Biometry of *nud* Knockout and *win1* Knockout Barley Lines under Ionizing Radiation

**DOI:** 10.3390/plants11192474

**Published:** 2022-09-22

**Authors:** Elena V. Antonova, Nadezhda S. Shimalina, Anna M. Korotkova, Ekaterina V. Kolosovskaya, Sophia V. Gerasimova, Elena K. Khlestkina

**Affiliations:** 1 Institute of Plant and Animal Ecology, Ural Branch of Russian Academy of Sciences, 8 Marta 202, 620144 Ekaterinburg, Russia; 2 N.I. Vavilov All-Russian Institute of Plant Genetic Resources, Bolshaya Morskaya 42-44, 190000 Saint-Petersburg, Russia; 3 Institute of Cytology and Genetics, Siberian Branch of Russian Academy of Sciences, Lavrentjeva 10, 630090 Novosibirsk, Russia

**Keywords:** *Hordeum vulgare*, CRISPR/Cas, *NUD*, *WIN1*, knockout line, radiation, seedling

## Abstract

The genes *NUD* and *WIN1* play a regulatory role in cuticle organization in barley. A knockout (KO) of each gene may alter plant mechanisms of adaptation to adverse environmental conditions. A putative pleiotropic effect of *NUD* or *WIN1* gene mutations in barley can be assessed in a series of experiments in the presence or absence of a provoking factor. Ionizing radiation is widely used in research as a provoking factor for quantifying adaptive potential of living organisms. Our aim was to evaluate initial stages of growth and development of barley lines with a KO of *NUD* or *WIN1* under radiation stress. Air-dried barley grains with different KOs and wild-type control (WT) were exposed to γ-radiation at 50, 100, or 200 Gy at a dose rate of 0.74 R/min. Approximately 30 physiological parameters were evaluated, combined into groups: (1) viability, (2) radiosensitivity, and (3) mutability of barley seed progeny. Seed germination, seedling survival, and shoot length were similar among all barley lines. Naked *nud* KO lines showed lower weights of seeds, roots, and seedlings and shorter root length as compared to *win1* KO lines. The shoot-to-root length ratio of *nud* KO lines’ seedlings exceeded that of *win1* KO and WT lines. In terms of the number of seedlings with leaves, all the KO lines were more sensitive to pre-sowing γ-irradiation. Meanwhile, the radioresistance of *nud* KO lines (50% growth reduction dose [RD_50_] = 318–356 Gy) and WT plants (RD_50_ = 414 Gy) judging by seedling weight was higher than that of *win1* KO lines (RD_50_ = 201–300 Gy). Resistance of *nud* KO lines to radiation was also demonstrated by means of root length (RD_50_ = 202–254 Gy) and the shoot-to-root length ratio. WT seedlings had the fewest morphological anomalies. In *nud* KO lines, mainly alterations of root shape were found, whereas in *win1* KO lines, changes in the color and shape of leaves were noted. Thus, seedlings of *nud* KO lines are characterized mainly by changes in the root system (root length, root number, and root anomalies). For *win1* KO lines, other parameters are sensitive (shoot length and alterations of leaf shape and color). These data may indicate a pleiotropic effect of genes *NUD* and *WIN1* in barley.

## 1. Introduction

Life on Earth has evolved under exposure to complex abiotic and biotic factors. Initially, natural background radiation (NBR)—caused by cosmic radiation and the decay of natural radionuclides scattered in the environment—was very high [1]. NBR intensity has gradually decreased due to the formation of the ozone layer, which has weakened the effects of cosmic radiation, and due to partial decay of radionuclides [1]. Over millions of years, under conditions of continuous action of ultra-low doses of ionizing radiation, the biota has adapted to the established NBR [2]. A decline of the level of natural radiation can lead to the suppression of bacterial growth, the formation of a stress response [3], a decrease in protein translation, an increase in the transport of substrates through the plasma membrane [4], higher sensitivity to subsequent action of alkylating compounds [5], and a change in the activity of antioxidant enzymes and in the frequency of spontaneous mutations in Chinese hamster cells [6]. Therefore, the background level of radiation is an integral part of the modern biota environment. NBR nowadays can be enhanced by a release of artificial radionuclides into the environment for example as a result of nuclear-weapon tests or regular or accidental releases and emissions in the nuclear industry [7].

Some of the first experiments assessing the impact of ionizing radiation on plants [8,9] were conducted shortly after the discovery of a new type of radiation or X-rays in 1895 by W. C. Röntgen [10]; a stimulatory influence of irradiation on seed germination and plant growth was reported [8,9]. In subsequent decades, data on radiation effects, including those at low doses, had accumulated further [11]. Data on the radioresistance of dormant seeds of more than 500 plant species and 200 intraspecific forms (subspecies, cultivars, and varieties) depending on the radiation type and irradiation regime have been summarized [12] because one of the important tasks of radiobiology is the comparative analysis of radioresistance of plants belonging to different taxa [13,14,15] characterized by means of evolutionary age and genome size [16].

The second important aim of plant radiobiology is the use of ionizing radiation for agriculture [17,18] because hormesis [19] and various mutations [14] and radiomorphoses [20,21,22] have been discovered. In natural ecosystems and agrocenoses, chronic exposure can act as a major environmental factor [20,21,22,23,24].

The radiosensitivity of an organism depends on a number of parameters that characterize different types of variation: e.g., biochemical, genetic, ontogenetic, populational, species, ecological, geographical, spatial, and temporal [2]. Under conditions of chronic and acute irradiation, the radiosensitivity of plants can be modified by other environmental factors [25,26]. Altering the sensitivity of organisms to radiation is especially important now due to intensive development of the nuclear industry and climate change [27].

Barley as a model object of research is widely used in radiobiology [28,29,30,31]. This species has been selected by the International Commission on Radiological Protection as one of references in the “wild grasses” group to assess the impact of chronic ionizing radiation on terrestrial ecosystems [32].

The cuticle, which is composed of cuticular lipids and epicuticular wax, plays an important role in plant resistance to adverse environmental conditions. The cuticle protects plants from ultraviolet radiation [33], extreme temperatures [34], pathogens [35,36], salinity [37], dehydration [38], and drought [39]. This tissue is considered a key evolutionary acquisition of terrestrial plants, and its properties are related to the traits important for crop breeding [40].

Both *NUD* and *WIN1* in barley belong to the WAX INDUCER/SHINE subfamily of genes encoding APETALA2/ETHYLENE RESPONSE FACTORS [41]. The known functions of these genes are related to lipid accumulation during different developmental stages. The product of the *NUD* gene controls the formation of a cementing layer between the pericarp and both lemmas (lower bracts) and palea (upper bract). A naturally occurring deletion of 17 kb at the barley (*Hordeum vulgare*) *NUD* (*HvNUD*) locus is associated with the appearance of naked barley [41]. The *Arabidopsis thaliana Win1* (*AtWIN1*) gene governs the accumulation of cuticular wax [42]. The barley plants mutant for this gene’s homolog, *HvWIN1*, are characterized by a deficiency of epicuticular wax on the surface of leaf sheaths and stems at the heading stage [43]. Overexpression of *AtWIN1* in *A. thaliana* raises the level of cutin and induces wax biosynthesis genes [44]. There is no evidence for the role of *HvWIN1* in cutin biosynthesis, but based on the homology to *AtWin1*, such a function is expected.

Cuticle properties in seeds and young seedlings may be crucial for resistance to both abiotic and biotic stressors. There is no evidence for the involvement of genes *NUD* and *WIN1* in cuticle and cuticle wax properties during early stages of plant development. Therefore, the purpose of this study was to search for pleiotropic effects of mutations in *HvNUD* or *HvWIN1*, which may affect cuticle organization in seeds and seedlings and barley resistance to various adverse conditions. Previously generated *nud* knockout (KO) and *win1* KO lines [41,43] were chosen here as a model for early-development assessment. We hypothesized that the *nud* KO and *win1* KO barley lines would perform differently in terms of growth characteristics and responses to ionizing radiation in comparison to each other and a wild-type control (WT) line.

## 2. Results

### 2.1. General Phenotyping of nud KO Lines

This procedure did not reveal any influence of a *HvNUD* gene mutation on major growth characteristics of plants besides grain weight. The absence of the hull caused losses in grain weight that are comparable with hull weight. Average values of parameters evaluated in three independent *nud* KO lines are given in Table 1. The same parameters have been measured in three independent *win1* KO lines, and no specific difference from the WT has been found [43]. No pleiotropic effects of the *HvNUD* and *HvWIN1* gene mutations on major growth performance characteristics were detectable here under normal conditions.

### 2.2. Thousand-Grain and Seedling Weights

**Figure 1:**
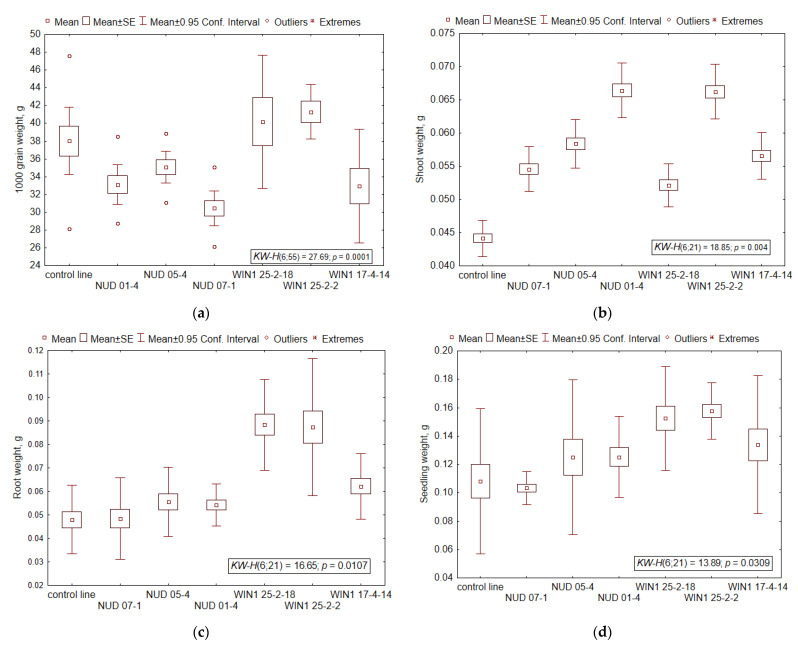
Variation of air-dried weights of grains and seedlings of *nud* KO and *win1* KO barley lines: (**a**) grains (number of samples for each line, *N* = 4–10), (**b**) shoots (*N* = 3), (**c**) roots (*N* = 3), and (**d**) seedlings (*N* = 3). The mean values, standard error (SE) and 95% confidence interval are indicated in the Figure 1. The nonparametric Kruskal-Wallis (KW) method was used, where *H* is the criterion value, *p*-value is the significance level. The degree of freedom (*df*) values of the numerator and denominator of *H*-criterion are given in brackets.

The weight of barley grains (Figure 1a) was found to be similar among different *nud* KO lines (*p* = 0.089–0.605), while *win1 17-4-14* has the lowest values (*p* = 0.006). In most cases, the grain weight of *nud* KO lines is lower than that of *win1* KO lines (Duncan’s pairwise comparison, *p* = 0.0001–0.006) and is in the range of the control line’s variation (the effect of the “line” factor was significant, *H*_2;21_ = 9.68; *p* = 0.0079). A similar relation was noted for root weight (Figure 1c) and seedling weight (Figure 1d). These patterns were confirmed by the correlation coefficients obtained via a comparison of seed and root weights (*R* = 0.58; *p* = 0.005) or grain and seedling weights (*R* = 0.45; *p* = 0.039). At the same time, the KO lines significantly exceed the WT values in shoot weight (Figure 1b) (Duncan’s pairwise comparison, *p* = 0.000029–0.000181), and there are differences between the lines of the same gene (*nud* KO: *p* = 0.00004–0.008; *win1* KO: *p* = 0.00004–0.0029). Differences among KO lines are significant only in pairs *win1 KO 25-2-2* versus *nud* KO *07-1* (*p* = 0.000065), *win1 KO 25-2-2* versus *nud* KO *05-4* (*p* = 0.000183), *win1 KO 25-2-18* versus *nud* KO *05-4* (*p* = 0.000248), *win1 KO 25-2-18* versus *nud* KO *01-4* (*p* = 0.000032), and *win1 KO 17-4-14* versus *nud* KO *05-4* (*p* = 0.000066).

### 2.3. Viability of Seed Progeny

**Figure 2:**
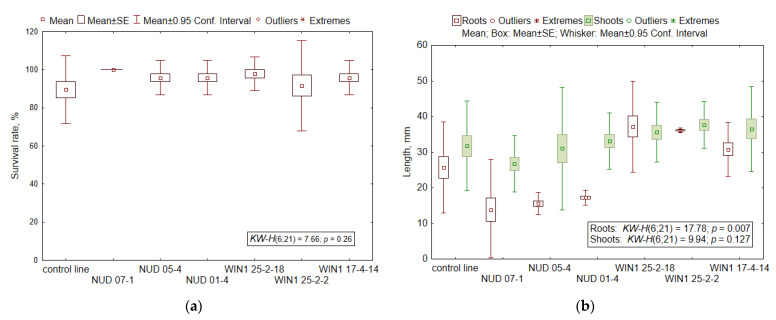
Viability indicators in seedlings of barley KO lines. (**a**) The survival rate, % (number of samples for each line, *N* = 3); (**b**) shoot (*N* = 43–48) and root lengths (*N* = 44–48), mm. The mean values, standard error (SE) and 95% confidence interval are indicated in the Figure 2. The nonparametric Kruskal-Wallis (KW) method was used, where *H* is the criterion value, *p*-value is the significance level. The degree of freedom (*df*) values of the numerator and denominator of *H*-criterion are given in brackets.

According to the main indicators of the viability of seed progeny (seed germination, seedling survival, and the proportion of plants with leaves), *nud* and *win1 KO* barley lines do not differ from each other (*H*_6;21_ = 7.5–9.5; *p* = 0.146–0.277). Given that seed germination and seedling survival (*R* = 0.53; *p* = 0.0078) as well as seedling survival and the number of seedlings with leaves (*R* = 0.76; *p* = 0.00007) correlated, this article presents only data on seedling survival (Figure 2a).

In growth characteristics (Figure 2b), for example shoot length, the lines do not differ significantly from each other (*H*_6;21_ = 9.94; *p* = 0.127). This parameter positively correlated with the number of roots (*R* = 0.49–0.58; *p* = 0.0065–0.039), root length (*R* = 0.70–0.86; *p* = 0.0001–0.0381), shoot weight (*R* = 0.62; *p* = 0.0025), and root weight (*R* = 0.61; *p* = 0.0034).

The smallest root length was observed in *nud* KO lines (Duncan’s pairwise comparison, the difference from the WT: *p* = 0.002–0.014, and from *win1 KO* lines: *p* = 0.000032–0.00036), and the largest in *win1 KO* lines (the difference from the WT: *p* = 0.0032–0.1165). Overall, the “line” factor had a significant effect on the average root length in seedlings (*H*_6;21_ = 17.78; *p* = 0.0068) and on the total root system length (*H*_6;21_ = 17.73; *p* = 0.0069) and did not affect the average number (*H*_6;21_ = 10.62; *p* = 0.10) and the sum of all roots in seedlings (*H*_6;21_ = 11.98; *p* = 0.0623). A positive correlation was noted between the first–fifth root length and seedling survival (*R* = 0.56–0.78; *p* = 0.00007–0.0078), between the root number and root length (*R* = 0.75–0.82; *p* = 0.00002–0.00009), and among the root number, the total root system length, and root weight (*R* = 0.55–0.85; *p* = 0.000001–0.018).

Since root length in the seedlings of *nud* KO lines was found to be shorter than that in the other lines, and because shoot length does not differ among the seven lines, the ratio of shoot length to the sum of root lengths or to the average root length of the seedlings in *nud* KO lines is 1.63–1.76 times higher than WT values and 1.96–2.16 times higher than *win1* KO lines’ values. These ratios in *win1* KO lines reach 0.81–0.83 of the WT values.

Thus, specific features of *nud* KO lines manifested themselves as reduced mass of grains, roots, and seedlings as well as the average and total root length as compared to *win1* KO lines. High variation of shoot weight was noted, both between *win1* KO and *nud* KO lines and between lines with a common mutant gene. The ratios of shoot length to the average and total root length in the seedlings of *nud* KO lines turned out to be higher than those in *win1* KO lines and the WT.

### 2.4. Radioresistance of the Seed Progeny

**Figure 3:**
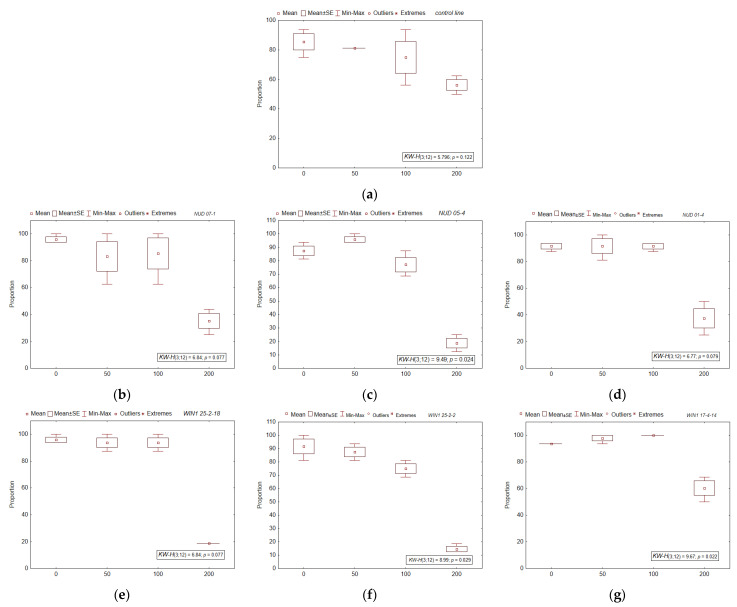
The proportion of seedlings with leaves in KO barley lines after irradiation. (**a**) WT, (**b**) *nud 07-1*, (**c**) *nud 05-4*, (**d**) *nud 01-4*, (**e**) *win1 25-2-18*, (**f**) *win1 25-2-2*, and (**g**) *win1 17-4-14*. The *x*-axis indicates radiation doses (Gy). The number of samples for each dose or line was *N* = 3. The mean, standard error (SE), minimum (min) and maximum (max) values are indicated in the Figure 3. The nonparametric Kruskal-Wallis (KW) method was used, where *H* is the criterion value, *p*-value is the significance level. The degree of freedom (*df*) values of the numerator and denominator of *H*-criterion are given in brackets.

Pre-sowing irradiation at doses of 50–200 Gy had no significant effect on seed germination and seedling survival in the seven lines (*H*_3;12_ = 0.24–6.73; *p* = 0.081–0.86). A decrease in the number of seedlings with leaves occurred only after irradiation at a maximum dose of 200 Gy (Duncan’s pairwise comparison, *p* = 0.000004–0.016). The most radiosensitive (Figure 3) were *nud 05-4*, *win1 25-2-18*, and *win1 25-2-2* KO lines: the number of seedlings with leaves decreased by 78.6–84.1% as compared to a respective non-irradiated control. The WT line and *win1 17-4-14* proved to be the most radioresistant (the decrease was 34.1–35.6% as compared to a respective non-irradiated control). This finding was confirmed by means of coefficients *b*_1_ of linear regression equations (the slope of the regression), which in *nud* KO and *win1* KO lines exceeded the WT values by 1.9–2.7-fold, with the exception of *win1* 17-4-14 (*b*_1_ exceeded the WT value by 1.2-fold).

**Figure 4:**
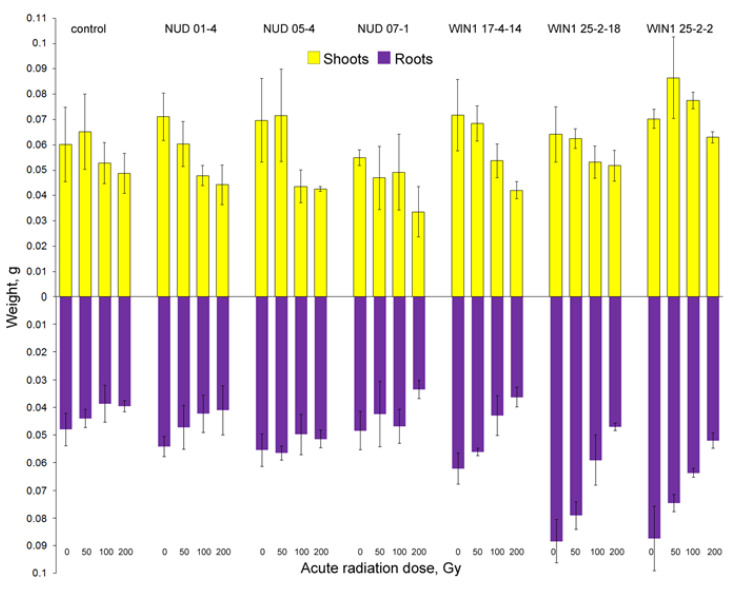
Weights of aboveground and underground parts of barley seedlings of KO lines after irradiation. The number of samples for each dose or line was *N* = 3.

The minimum irradiation dose of 50 Gy had no effect on shoot and root weights and seedlings (Figure 4) in all lines (Duncan’s test, *p* = 0.133–0.99). Similar results were obtained after irradiation at 100 Gy for shoot weight in all the tested lines (*p* = 0.063–0.62); a significant decline of this parameter was observed only in the *nud 05-4* line (*p* = 0.0375). After irradiation at 200 Gy, a significant decrease in this parameter was noted in *nud 05-4*, *nud 01-4*, and *win1 17-4-14* lines (*p* = 0.018–0.03). Root weight after 100 Gy irradiation diminished only in *WIN1* line seedlings (*p* = 0.000005–0.0071), whereas at 200 Gy, it decreased in *nud 07-1* (*p* = 0.000004–0.039) too. After irradiation at 100 or 200 Gy, seedling weight normalized to group size declined in all the studied lines (*p* = 0.0001–0.004), except for the WT and the *nud 07-1* KO line (*p* = 0.33–0.64). Values of *b*_1_ coefficients of the linear regression equations (Table 2) for *win1* KO lines exceeded the WT values by 1.8–2.8-fold. Overall, weight characteristics of *win1* KO lines were more sensitive to irradiation relative to *nud* KO lines and WT plants.

**Figure 5:**
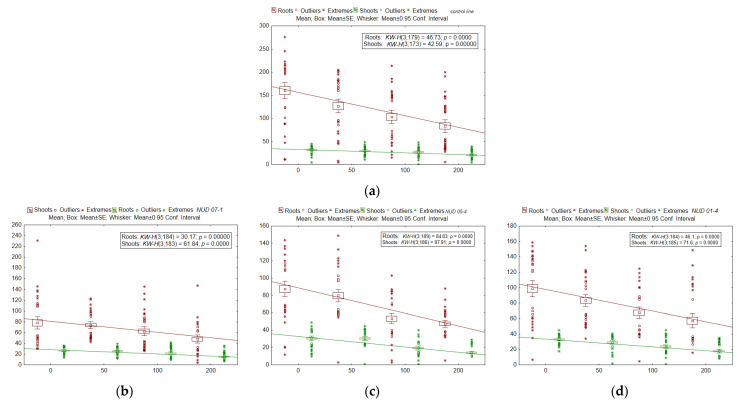

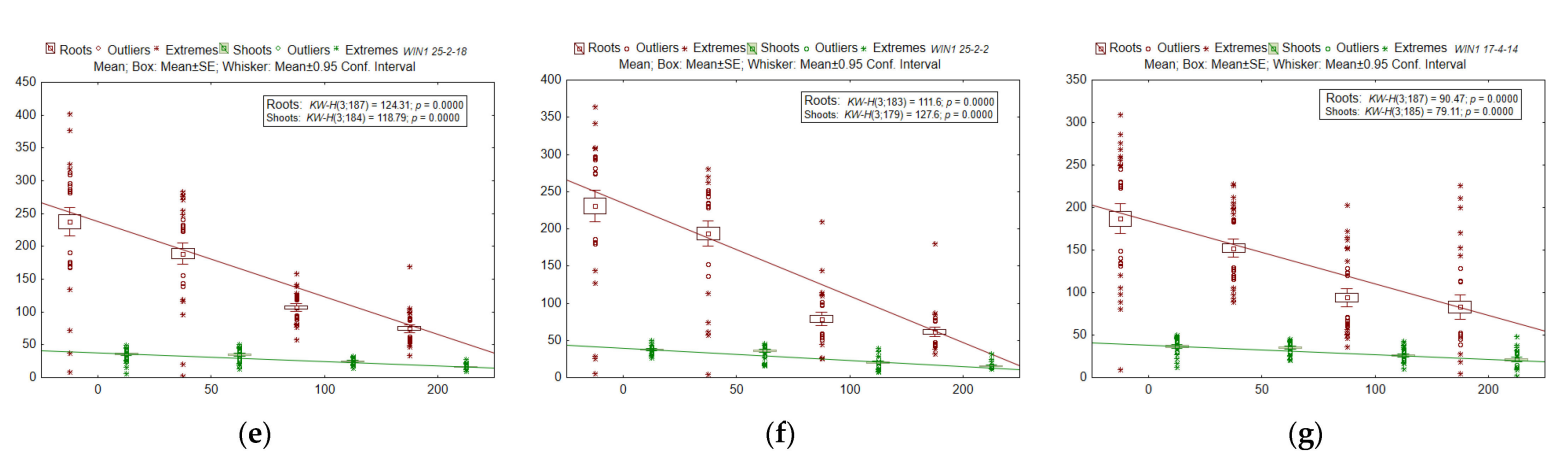
The average shoot length (mm) and the total root length (mm) in seedlings of barley KO lines after irradiation. (**a**) WT, (**b**) *nud 07-1*, (**c**) *nud 05-4*, (**d**) *nud 01-4*, (**e**) *win1 25-2-18*, (**f**) *win1 25-2-2*, and (**g**) *win1 17-4-14*. The *x*-axis denotes radiation doses (Gy). The number of samples for each dose or line was *N* = 41–48 (shoots) and *N* = 43–48 (roots). The mean, standard error (SE) and 95% confidential interval are indicated in the Figure 5. The nonparametric Kruskal-Wallis (KW) method was used, where *H* is the criterion value, *p*-value is the significance level. The degree of freedom (*df*) values of the numerator and denominator of *H*-criterion are given in brackets.

An analysis of the growth characteristics showed (Figure 5) that after irradiation at the minimum dose of 50 Gy, shoot length did not change in seedlings of any lines (*p* = 0.67–0.87). The maximum dose of 200 Gy significantly reduced this parameter (*p* = 0.000004–0.017). Similar data were obtained after irradiation at 100 Gy (*p* = 0.00013–0.043), except for the control line (a decrease by 14.9%) and *nud 07-1* (by 19.4%); the differences were insignificant (*p* = 0.25–0.31). Just as in terms of the “number of seedlings with leaves,” the WT was the most resistant to irradiation judging by shoot length (at the maximum dose: a 34.9% decline relative to the non-irradiated control), and *win1* KO lines were the most sensitive (a 41.5–58.5% drop in comparison with the non-irradiated control). This finding was confirmed by *b*_1_ coefficients from the linear regression equations (see Table 2), which for *win1* KO lines exceeded the WT values by 1.4–2.1-fold.

The root number (mean: *p* = 0.125–0.92, and total: *p* = 0.144–0.91) in seedlings of isogenic lines did not change after irradiation, except for the *nud 07-1* KO line (*p* = 0.009–0.017). For instance, after irradiation at 200 Gy, the seedlings of this line showed a decrease in the root number of seedlings to 4.9 ± 0.16 as compared with their non-irradiated control (5.54 ± 0.11). Root length of seedlings was more sensitive to irradiation in the entire tested dose range (*p* = 0.000001–0.017). The exception was all *nud* KO lines, in which after irradiation at 50 Gy, there was no significant decline of the total root system length (*p* = 0.113–0.648) and of the average root length in seedlings (*nud 07-1*: *p* = 0.59; *nud 05-4*: *p* = 0.056). In addition, the *nud 07-1* line did not experience a decrease in the total root length even after irradiation at 100 Gy (*p* = 0.109). At the maximum dose, the radioresistance was comparable between the WT and *nud* KO lines (52.2–58% of the non-irradiated control) and exceeded that of *win1* KO lines (34.1%). Coefficients *b*_1_ of the linear regression equations (see Table 2) for *win1* KO lines exceeded the WT values by 1.5–2.4-fold, and for *nud* KO lines, they amounted to 40–60% of the WT values, indicating the stability of *nud* KO lines.

Since root length after irradiation diminished more strongly than did shoot length in seedlings, an increase in the ratio of shoot length to the total root length was observed in the WT (115.8–127.4%) and *win1* KO (109.8–171.8%) lines (*p* = 0.000001–0.017). No change in the ratios of these growth parameters was observed in *nud* KO lines after irradiation at 50–100 Gy (*p* = 0.179–0.84); the length of seedling roots was still shorter in *nud* KO lines than in the other lines. Only at the maximum dose of 200 Gy was there a decrease in this parameter in *nud 07-1* and *nud 05-4* lines, by 13.1–20.4% relative to a respective non-irradiated control (*p* = 0.000096–0.011) owing to shortening of the shoots. The *nud 01-4* line is characterized by the stable ratio of shoots to roots across the entire range of irradiation doses (*p* = 0.195–0.79).

Overall, γ-irradiation had a significant impact on the most sensitive processes: cell division (thereby leading to a decrease in the weight of seedlings), the number of seedlings with leaves, and shoot and root lengths; there was no influence on seed germination, seedling survival, and the root number. Radioresistance—as estimated with the help of sensitive indicators—differed among the analyzed lines. In terms of the number of seedlings with leaves, the WT line was the most resistant to irradiation. At the same time, judging from the weights of roots and seedlings, the radioresistance of *nud* KO lines and WT plants was higher than that of *win1* KO lines. *Nud* KO lines were also found to be radioresistant in terms of root length and the shoot-to-root length ratio.

### 2.5. Mutability of the Seed Progeny

The number of seedlings with anomalies was the smallest in the WT line (Table S1): only an alteration of leaf color (Figure S1a) was detectable (in 6.7% of plants) and hairiness of roots (13.3%, Figure S1b). *Nud* KO lines manifested mainly root anomalies. For instance, a change in the shape of roots (Figure S1c) was observed in 10.3–16% of these plants, whereas the “dancer” anomaly (Figure S1d) and pubescent roots were observed in almost all these plants. The occurrence of yellow-green leaves varied from 6.7% to 13.3% of seedlings (see Table S1). At the same time, color anomalies (prevalence 15.1–63.1%) and leaf shape aberrations (12.5–100%, Figure S1e) were predominant in *win1* KO lines. Alterations of the shape of the coleoptile (twisting (Figure S1f) and tearing (Figure S1g) due to incorrect leaf exit) were rare and were documented only in *nud 01-4* (prevalence 6.7%) and *win1 17-4-14* (6.3%). Root necrosis negatively correlated with the length, number, and mass of roots (*R* = −0.699 to −0.988; *p* = 0.000001–0.041) and with changes in their shape (*R* = –0.99; *p* = 0.002). There was an opposite relation between the length, number, and weight of roots and leaf color alterations in seedlings (*R* = 0.58–0.96; *p* = 0.015–0.047).

After irradiation at different doses, the variety of anomalies increased. For example, the emergence of twins (6.7%, Figure S1h) was found in the *win1 25-2-18* line, but it was random. Hairiness of roots after irradiation increased 3.2–6.8-fold in the WT line, whereas this increase in *nud* KO lines was 0.33–7.96-fold, and in *win1* KO lines, it was only 0.4–1.52-fold (see Table S1). We also noted that other root anomalies, such as necrosis (Figure S1d,i), were also common in *nud* KO lines after irradiation (prevalence 82.2–100%) and were rare in *win1* KO lines (0–12.5%); the prevalence in the WT was 7.5–13.1%. Meanwhile, *win1* KO lines, just as before irradiation, had leaf shape anomalies (6.3–12.5%), which were not detectable in *nud* KO lines and in the WT.

## 3. Discussion

A general evaluation of the *win1* KO and *nud* KO lines’ growth performance has not revealed significant differences from the WT line [43]. This observation suggests that these genes perform a very specific function in plant development, and their mutations do not have a significant pleiotropic effect. Nevertheless, a knockout of the *NUD* gene leads to the naked grain phenotype, which obviously affects grain properties. Thus, the pleiotropic effect of the *NUD* gene mutation is expected for the grain and early development. On the contrary, a knockout of the *WIN1* gene does not exert visible effects on the grain, except for the deficiency of cuticle wax on the surface of the lemma [43]. Whether a deficit of surface wax influences grain properties is unknown.

Previously, it has been thought that different alleles of the *NUD* locus might affect the viability of seedlings. Nonetheless, the absence of the hull does not have an epistatic effect on other loci that control this trait [45]. In the present work, we documented similar dependences: seed germination, seedling survival, and shoot length each proved to be similar among *nud* KO, *win1* KO, and WT lines. Our results and available publications indicate that nakedness is not associated with growth performance, heading time, maturity, resistance to smut and burns, and ear density [46]. According to our data (Table 3), specific features of the naked *nud* KO lines manifested themselves as reduced weights of seeds, roots, and seedlings as compared to *win1* KO lines, as well as lesser root length. The shoot-to-root length ratio in seedlings of *nud* KO lines exceeded that of *win1* and WT lines (see Table 3). Similar data were obtained in a report that showed a correlation between nakedness and low plant weight, height, 1000-seed weight, and yield [46]. The difference in grain weight can be attributed to the loss of the hull and usually matches hull weight, which is estimated at 10–13% [46]. Other differences, especially suppressed root development, may be caused by damage to or stress on the unprotected embryo.

We demonstrated that pre-sowing γ-irradiation had a significant influence on the most sensitive processes: cell division (thereby diminishing the weight of seedlings), the number of seedlings with leaves, shoot and root lengths, and the number of roots (Table 3); there was no effect on seed germination and seedling survival. These findings are in good agreement with the data published in ref. [31]: γ-irradiation of seeds at 100 Gy causes significant growth inhibition and contributes to changes in the amounts of transcripts associated with cell cycle arrest, DNA damage repair, and the antioxidant system.

According to the number of seedlings with leaves, all KO lines are more sensitive to irradiation than the WT is. *Win1* KO lines proved to be the most sensitive in terms of weights of roots and seedlings and shoot length. These lines’ 50% growth reduction dose (RD_50_), as determined via shoot length, is 158–225 Gy, which is less than WT values (285 Gy) obtained in this experiment and reported for seven barley varieties (330–450 Gy) described in ref. [29]. *Nud* KO lines can be distinguished by low radioresistance judging from shoot weight and the root number in our work. Nonetheless, they turned out to be radioresistant as evidenced by root length and the shoot-to-root length ratio. RD_50_ for root length is 202–254 Gy for *nud* KO lines, which is comparable to the lower limit of 250–400 Gy published elsewhere [29]. The reason for inhibition of root growth may be an increase in the cell wall rigidity associated with the formation of oxidative crosslinks in the apoplast and a decrease in intraroot osmotic pressure owing to a limited supply of assimilates from irradiated leaves [47].

The WT line has the lowest number of seedlings with anomalies (see Table S1). In *nud* KO lines, mainly alterations of the shape of roots were registered here, such as hairy roots and the “dancer” phenotype. The latter anomaly has previously been described by us in seedlings of *Triticum aestivum* grown under adverse weather conditions [25]. According to our current findings, in the other KO lines (*win1* KO), anomalies of the color and shape of leaves are predominant.

## 4. Materials and Methods

### 4.1. Plant Models

Two-row spring barley (*Hordeum vulgare* L.) [48] cv. Golden Promise was used as donor material for targeted mutagenesis of genes *HvNUD* and *HvWIN1* by means of the Cas9/gRNA system [41,43]. An unmodified line of hulled barley cultivar Golden Promise served as a WT control line.

Using two guide RNAs (Nud45 and Nud50) for Cas9-based genome editing, we have previously obtained plants with various mutations in *HvNUD* [41] or *HvWIN1* [43].

In the second generations, plants with mutations of interest were selected to bring them to a homozygous state and remove transgenicity. Three homozygous lines of either the *nud* KO or *win1* KO genotype were selected for further experiments. The selected lines harbor the following mutations: deletion of position −3 nt (*nud50 01-4*), insertion at position +1 nt (*nud50 05-4*), and deletion of position −1 nt (*nud50 07-1*) in the *HvNUD* gene and a combined mutation including deletion −4 nt and insertion +10 nt (*win1* 17-4-14) and a single-nucleotide deletion (*win1* 25-2-2 and *win1* 25-2-18) in the *HvWIN1* gene. All selected lines possess the same phenotype consistent with the gene mutated. All *nud* KO lines have the naked grain, and all *win1* KO lines are deficient in epicuticular wax.

### 4.2. Greenhouse Growth Conditions

The plants were grown in the greenhouse complex of the Institute of Cytology and Genetics (ICG), the Siberian Branch of the Russian Academy of Sciences (SB RAS) (Novosibirsk, Russia), at the multi-access center Laboratory of Artificial Plant Cultivation at 20–25 °C under a 12 h photoperiod at ~25,000 lux illumination until mature seeds formed. Halogen lamps were used as a light source.

### 4.3. General Phenotyping of nud KO Lines

Greenhouse-grown mature plants were collected. The following parameters were measured: the total number of tillers and the number of fertile tillers per plant, height of the main tiller, length of the main spike, the spikelet number of the main spike, the grain number of the main spike, grain weight of the main spike, 1000-grain weight, the grain number per plant, and the grain yield per plant. Ten to 20 air-dried barley seeds were weighed in 4–10 replicates on an analytical balance (Kern 770, Balingen, Germany). The weight was calculated per 1000 grains.

### 4.4. Pre-Sowing Seed Treatment

The radioresistance of barley lines was assessed under laboratory conditions. Irradiation doses for mature and dormant seeds were chosen according to LD_50_ of various barley cultivars [12,14,15], excluding the range of low doses (up to 20 Gy), at which hormesis can take place [28]. Irradiation of air-dried seeds was carried out on a γ-installation of the “Researcher” type with a ^137^Cs source at a dose rate of 0.74 R/min and doses of 50, 100, or 200 Gy (Department of Radioecology, federal research center of the Institute of Biology of Komi Science Centre (IB KSC), the Ural Branch of the Russian Academy of Sciences (UB RAS) (Syktyvkar, Russia).

### 4.5. Experimental Design

To assess viability, radiosensitivity, and mutability of seed progeny of the barley KO lines, into disposable sterile plastic Petri dishes with a cotton filter, 10 mL of distilled water was added, and 16 seeds were sown in each, arranged in the 4 × 4 seed pattern. The Petri dishes were sealed with adhesive tape to prevent water evaporation. The experiments were performed on three biological replicates (the total number of seeds: 1344).

### 4.6. Seedling Emergence Rates and Growth Characteristics

The first 2 days of germination were synchronized via keeping of the seeds (planted in Petri dishes) at 6 °C in the dark. Next, the seeds were germinated in a climate room of the Institute of Plant and Animal Ecology (IPAE), the Ural Branch of the Russian Academy of Sciences (UB RAS) (Ekaterinburg, Russia), at 20 °C under a 12 h photoperiod.

The Petri dishes were randomized daily. Five days after the sowing of the seeds, more than 30 parameters were quantified (seed germination, seedling survival, the number of seedlings with leaves, the length of shoots, the average number of roots, lengths of 1–11 roots [root test], the weight of shoots, the weight of roots, alterations of the shape of roots, root pubescence, necrosis, changes in the color and shape of leaves, alterations of coleoptile shape, and the appearance of twins). In addition, we calculated the total length of the root system, the average length of the roots of seedlings, and the shoot-to-root length ratio. Indicators of the quality of seed progeny served as the main indicators of viability. Radiosensitivity was determined according to the criteria described above, in relative units toward a respective non-irradiated control.

### 4.7. Data Analysis

At the end of the experiment, seedlings were left in the dark to reduce growth. The obtained values of the growth parameters of the seedlings were normalized to the time points of measurements in order to minimize variance among replicates and different lines. Statistical hypotheses were assessed by the asymptotic two-sided test for a difference between two proportions, by the parametric Fisher test (*F*), nonparametric Mann–Whitney (*U*) and Kruskal–Wallis (*H*) tests, ANOVA and MANOVA, Duncan’s multiple comparison tests, and by correlation (*R*) and regression analyses. The normality of data distribution was evaluated by the Kolmogorov–Smirnov test (*d*) with Liliefors and Shapiro–Wilks (*W*) corrections. The calculations were carried out in STATISTICA 10.0 [49] (Tulsa, OK, USA) and Past 2.11 [50] software.

## 5. Conclusions

The study revealed pleiotropic effects of knockouts of genes *HvNUD* and *HvWIN1*. The pleiotropic effects of the *HvNUD* gene knockout include the appearance of short roots in barley seedlings, a decrease in the root number after irradiation, the emergence of root anomalies, and root necrosis. The pleiotropic effects of the *HvWIN1* gene manifested themselves as higher viability, the appearance of leaf aberrations (shape and color), and the sensitivity of shoot length to ionizing radiation. Overall, the radioresistance of the analyzed barley lines can be ranked as follows: WT ≥ *nud* > *win1*.

The investigation into the influence of ionizing radiation on *nud* and *win1* KO barley lines allows us to hypothesize their tolerance to other abiotic factors. The assessment of stress responses of *nud* and *win1* KO lines at the seedling stage makes it possible to predict resistance to these stressors at late stages of plant development.

## Figures and Tables

**Table 1:** General phenotyping of the *nud* KO lines (mean ± standard deviation).

Trait	Control Line	*nud50* KO Lines		
	WT	*nud* 01-4	*nud* 05-4	*nud* 07-1
Spikelet number per spike	11.8 ± 0.63	12.2 ± 0.63	11.6 ± 1.27	12.4 ± 1.27
Spike length, cm	8.1 ± 0.53	7.3 ± 0.40 *	7.3 ± 0.33 *	7.3 ± 0.96
Main spike length, cm	8.7 ± 0.94	8.0 ± 0.78	8.7 ± 0.63	8.3 ± 1.44
Grain number per main spike	15 ± 3.68	14.8 ± 5.55	17.6 ± 1.5	20.3 ± 3.6 *
Grain number per plant	111.3 ± 33.8	119.1 ± 61.8	123.5 ± 67.9	109.4 ± 59.2
Grain weight per main spike, g	0.67 ± 0.19	0.52 ± 0.14	0.70 ± 0.17	0.71 ± 0.18
Grain weight per plant, g	4.12 ± 0.81	3.87 ± 1.80	4.35 ± 2.42	3.42 ± 1.96
1000-grain weight, g	38.0 ± 5.31	33.1 ± 3.14	35.1 ± 2.52	30.4 ± 2.73 *
Plant height, cm	62.5 ± 4.60	57.6 ± 3.27	60.4 ± 5.32	59.5 ± 5.38
Total number of tillers	9.1 ± 2.69	8.6 ± 4.27	9.2 ± 3.79	6.2 ± 2.97
Number of fertile tillers	8.0 ± 1.70	8.0 ± 3.65	7.8 ± 3.97	5.9 ± 2.96
Awn length, cm	9.3 ± 0.68	8.9 ± 0.81	9.05 ± 0.73	9.1 ± 0.84

* Differences between KO and WT lines were significant (Mann–Whitney *U*-test, *p* = 0.013–0.027).

**Table 2:** Sensitivity of the seven barley lines to γ-radiation (linear regression coefficients *b*_0_ and *b*_1_ are given).

Line	Index	*b*_0_	*b*_1_	RD_50_, * Gy
WT	Seedling weight	0.0075	−9.05 × 10^−6^	414
*nud 07-1*		0.0066	−9.28 × 10^−6^	356
*nud 05-4*		0.0081	−1.25 × 10^−5^	324
*nud 01-4*		0.0077	−1.21 × 10^−5^	318
*win1 25-2-18*		0.0095	−1.58 × 10^−5^	300
*win1 25-2-2*		0.0102	−2.53 × 10^−5^	201
*win1 17-4-14*		0.0084	−1.63 × 10^−5^	258
WT	Total root length	150.6	−0.37	206
*nud 07-1*		79.4	−0.16	254
*nud 05-4*		85.5	−0.21	202
*nud 01-4*		94.8	−0.21	231
*win1 25-2-18*		225.5	−0.83	135
*win1 25-2-2*		220.1	−0.90	123
*win1 17-4-14*		175.4	−0.53	165
WT	Shoot length	32.4	−0.06	285
*nud 07-1*		27.7	−0.06	224
*nud 05-4*		31.8	−0.09	173
*nud 01-4*		32.7	−0.08	212
*win1 25-2-18*		36.8	−0.10	178
*win1 25-2-2*		37.9	−0.12	158
*win1 17-4-14*		36.9	−0.08	225

* 50% growth reduction dose.

**Table 3:** Comparative evaluation of significant differences among barley KO lines in terms of viability, radiosensitivity, and seed mutability.

Parameters	Lines		Resume
	*nud*	*win1*	
Viability			
1000-grain weight	≈WT, <*win1*	>WT, >*nud*	>*win1*
Root weight	≈WT, <*win1*	>WT, >*nud*	>*win1*
Shoot weight	>WT, <*win1*	>WT, >*nud*	>*win1*
Seedling weight	≈WT, <*win1*	>WT, >*nud*	>*win1*
Root length	<WT, <*win1*	>WT, >*nud*	<*nud*
Shoot/Root length	>WT, >*win1*	≈WT, <*nud*	>*nud*
Radioresistance			
Seedlings with leaves	<WT, ≈*win1*	<WT, ≈*nud*	all KO lines are sensitive
Root weight	<WT, >*win1*	<WT, <*nud*	*win1* is more sensitive
Shoot weight	<WT, <*win1*	<WT, >*nud*	*nud* is more sensitive
Seedling weight	<WT, >*win1*	<WT, <*nud*	*win1* is more sensitive
Shoot length	<WT, >*win1*	<WT, <*nud*	*win1* is more sensitive
Root number	<WT, <*win1*	≈WT, >*nud*	*nud* is more sensitive
Root length	>WT, >*win1*	<WT, <*nud*	*nud* is more resistance
Shoot/Root length	>WT, >*win1*	≈WT, <*nud*	*nud* is more resistance
Morphological abnormalities			
Root shape change	>WT, >*win1*	>WT, <*nud*	common in *nud*
Leaf color change	>WT, <*win1*	>WT, >*nud*	common in *win1*
Leaf shape change	>WT, <*win1*	>WT, >*nud*	common in *win1*
Coleoptile shape change	>WT, ≈*win1*	>WT, ≈*nud*	common in both KO lines
Hairy roots (R *)	<WT, >*win1*	<WT, <*nud*	common in WT
Root necrosis (R *)	>WT, >*win1*	>WT, <*nud*	common in *nud*

* after acute irradiation.

## Data Availability

Not applicaple.

## Supplementary Materials

The following supporting information can be downloaded at: https://www.mdpi.com/article/10.3390/plants11192474/s1, Figure S1: Morphological anomalies found in *H. vulgare nud* KO and *win1* KO lines: a—yellow-green leaf, b—hairiness of roots, c—twisting root, d—‘dancers’ with root necrosis, e—twisting and yellow-green leaf, f—twisting coleoptile, g—torn coleoptile, h—twins, i—root necrosis, thickening and twisting; Table S1: Variety of morphological anomalies in barley *nud* and *win1* KO lines without and after γ-irradiation (Mean ± S.E.).
