# Supplementary material for: Seedling Biometry of nud Knockout and win1 Knockout Barley Lines under Ionizing Radiation
Source: Plants (Basel). 2022 Sep 22;11(19):2474. doi: 10.3390/plants11192474 (PMC9571651; doi:10.3390/plants11192474)
Supplement: Supplementary file 1 [file plants-11-02474-s001.zip › plants-1869724-supplementary.pdf]

Supplementary Materials

Table S1. Variety of morphological anomalies in barley *nud* and *win1* KO lines without and after γ-irradiation (Mean ± S.E.).

Abnormality, %	WT	*nud* 07-1	*nud* 05-4	*nud* 01-4	*win1* 25-2-18	*win1* 25-2-2	*win1* 17-4-14
0 Gy							
Root shape	-	16±0	15.3±0.3	10.3±4.2	-	-	-
Hairiness of roots	13.3±0	37.5±25	18.9±12.3	-	66.7±0	65.63±34.4	100±0
Dancer	-	100±0	100±0	100±0	-	-	-
Root necrosis	-	95.8±4.2	97.9±2.1	97.8±2.2	-	12.5±0	-
Leaf color	6.7±0	-	13.3±0	6.7±0	63.1±21.2	46.1±7.4	15.1±1.8
Leaf shape	-	-	-	-	100±0	-	12.5±0
Coleoptile shape	-	-	-	6.7±0	-	-	6.3±0
50 Gy							
Root shape	-	9.7±4.5	15.3±0.3	15.3±0.7	2±0	-	-
Hairiness of roots	42.6±26	12.5±6.3	39.8±33.5	6.7±0.5	26.7±20	67.9±32.1	100±0
Dancer	-	100±0	100±0	100±0	-	-	-
Root necrosis	10.7±3.6	100±0	100±0	100±0	-	-	12.5±0
Leaf color	-	6.3±0	-	7.7±0	37.5±12.6	31.4±6.6	9.4±3.1
Leaf shape	-	-	-	-	6.7±0	-	12.5±0
Coleoptile color	-	-	-	7.7±0	6.7±0	-	-
Coleoptile shape	-	-	-	-	6.7±0	-	-
Twins	-	-	-	-	6.7±0	-	-
100 Gy							
Root shape	2±0	16±0	15±0	10±4.5	-	-	1±0
Hairiness of roots	45.0±28.3	37.5±28.2	56.7±16.7	13.3±0	97.9±2.1	53.3±46.7	100±0
Dancer	-	100±0	100±0	100±0	100±0	100±0	-
Root necrosis	7.5±0.8	89.6±10.4	100±0	82.2±17.8	-	6.7±0	-
Leaf color	-	-	-	-	-	6.7±0	9.4±3.1
Leaf shape	-	-	-	-	6.3±0	-	6.3±0
200 Gy							
Root shape	-	8.0±7.0	11.0±4.5	10.7±4.8	-	1.0±0	1.0±0
Hairiness of roots	91.3±5.9	53.3±13.3	69.7±15.8	53.3±46.7	95.8±4.2	-	87.5±12.5
Dancer	-	100±0	100±0	100±0	100±0	100±0	53.6±46.4
Root necrosis	13.1±6.9	44.8±25.4	93.3±6.7	6.7±0	9.8±3.5	-	6.3±0
Leaf color	-	-	-	-	-	-	7.7±0
Leaf shape	-	-	-	-	-	-	7.7±0
Coleoptile shape	-	-	-	-	6.5±0.2	-	15.4±0

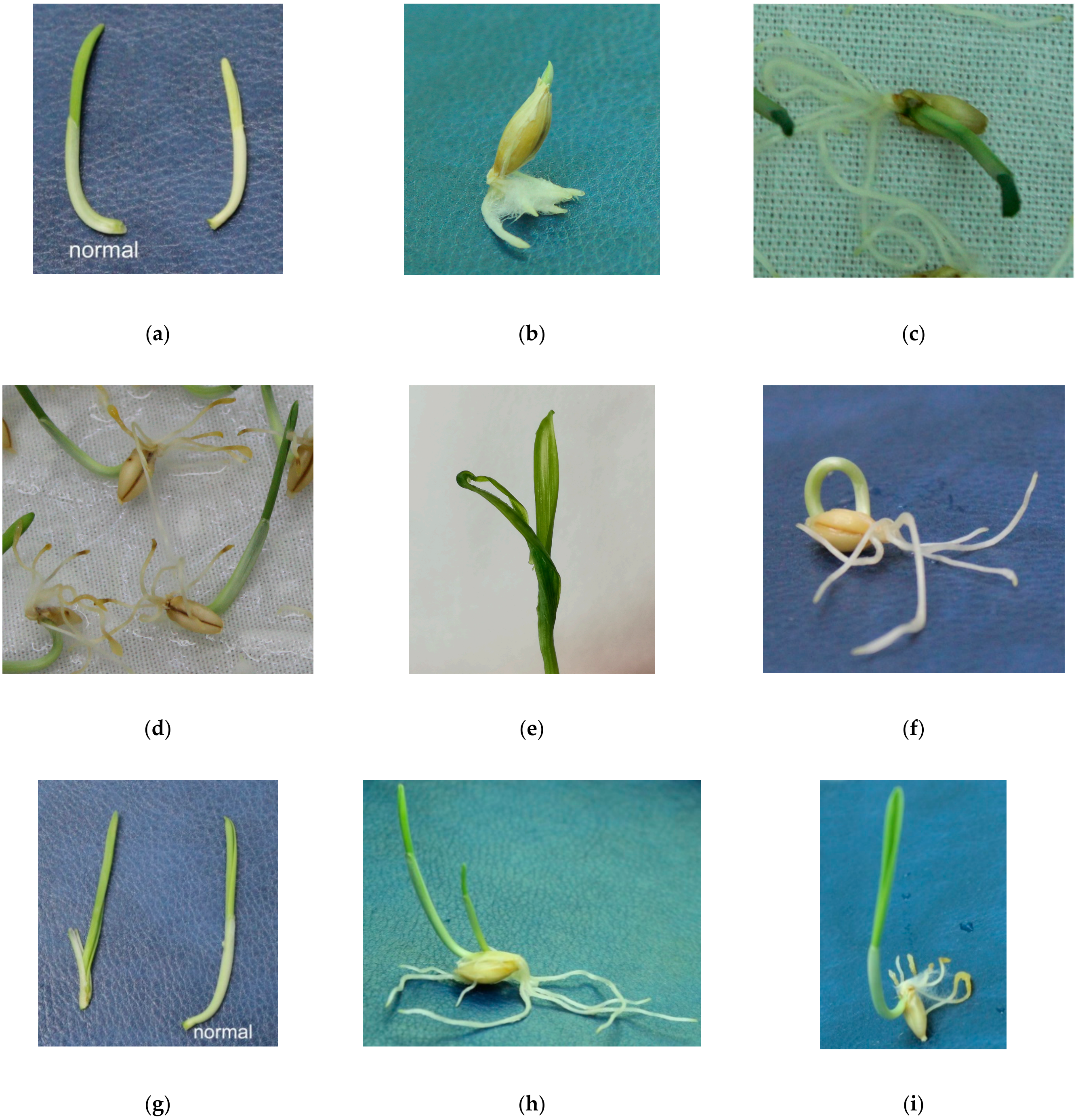

Figure S1. Morphological anomalies found in *H. vulgare nud* KO and *win1* KO lines: a – yellow-green leaf, b – hairiness of roots, c – twisting root, d – 'dancers' with root necrosis, e – twisting and yellow-green leaf, f – twisting coleoptile, g – torn coleoptile, h – twins, i – root necrosis, thickening and twisting.
